# Preparation and bacteriostatic research of porous polyvinyl alcohol / biochar / nanosilver polymer gel for drinking water treatment

**DOI:** 10.1038/s41598-021-91833-9

**Published:** 2021-06-09

**Authors:** Hang Zhao, Xuexiang Li, Liang Zhang, Zhihui Hu, Lvling Zhong, Juanqin Xue

**Affiliations:** 1 School of Chemistry and Chemical Engineering, Xi’an University of Architecture and Technology, Xi’an, 710055 Shaanxi China; 2 School of Chemistry and Chemical Engineering, Northwestern Polytechnical University, Xi’an, 710072 Shaanxi China; 3 Shannxi Provincial Key Laboratory of Gold and Resource, Xi’an University of Architecture and Technology, Xi’an, 710055 Shaanxi China

**Keywords:** Environmental sciences, Chemistry, Materials science

## Abstract

Microbial contamination in drinking water has become an important threat to human health. There is thus an urgent need to develop antibacterial materials to treat drinking water. Here, porous silver-loaded biochar (C–Ag) was prepared using corn straw as the substrate and silver as the antibacterial agent. C–Ag was then uniformly distributed in polyvinyl alcohol gel beads of eluted calcium carbonate to prepare p-PVA/C–Ag antibacterial composite. The polymer composites were tested by FT-IR, XRD, SEM and TG-DSC. The results showed that C–Ag was more evenly distributed in the PVA gel spheres. Antibacterial experiments showed that p-PVA/C–Ag greatly inhibited *Escherichia coli*. Practical application tests revealed that p-PVA/C–Ag showed high and sustained bactericidal inhibition and reusability. Generally, p-PVA/C–Ag composite shows high potential to be applied to drinking water treatment.

## Introduction

Clean, innoxious water is essential for life. Nowadays, an increasing number of industrial production and complex life activities have seriously affected the safety of water quality. Drinking water treatment requires more than simple filtration for ensuring that strict water quality testing standards are met; the removal of harmful microorganisms and heavy metals is also often needed. In the process of treating drinking water, untimely cleaning and disinfection of the water supply system can lead to the growth of pathogenic microorganisms such as bacteria and viruses^1^. Polluted water can also become a means by which diseases can be transmitted and pose a serious threat to the human population^2,3^. Furthermore, as society continues to develop and science and technology continue to advance, the level of industrialization in China continues to grow, and along with it the pollution of water resources, among which heavy metal pollution and pollution by microorganisms are the most serious problems requiring attention. Current standards of drinking water in China require the absence of pathogenic microorganisms in drinking water and specify that the contents of chemical substances in drinking water should adhere to strict limits (e.g., Cu^2+^ 1 mg L^−1^ and Pb^2+^ 0.01 mg L^−1^, GB5749-2006, China). There is thus a pressing need to address these issues, especially in developing countries.

Chlorination has been the main disinfection measure for drinking water used by most countries for many years. Although it is low-cost and has a strong disinfection effect, chlorination can cause serious organic pollution in water; specifically, hypochlorous acid, which is generated by the hydrolysis of chlorine disinfectant after being added to water, can easily oxidize Br^−^ in water, creating various disinfection by-products (DBPs) during the disinfection process. Ultraviolet disinfection is an environmentally friendly disinfection method that does not require the addition of chemicals and non-toxic by-product residues. However, the weak penetration of UV only permits the killing of bacteria, fungi and other microorganisms upon direct exposure. In practical applications, ultraviolet disinfection has been used as an auxiliary method in combination with other disinfection technologies, such as chlorination, to achieve a stronger disinfection effect^4,5^. Cuthbertson et al.^6^ showed that granular activated carbon can effectively reduce and remove DBP precursors and reduce the formation of DBPs but cannot completely eliminate them. Studies have shown that brominated DBPs are ordinarily more genotoxic, cytotoxic, developmentally toxic and growth-suppressing than their chlorinated analogs^7,8^. Adsorption and filtration are still the main methods for terminal drinking water treatment, and activated carbon is the most commonly used adsorbent. Physical adsorption and chemical adsorption of activated carbon adsorption can remove pollutants in water^9,10^. However, the treatment effect of activated carbon is limited. Su et al.^11^ showed that granular activated carbon filtration can even increase the content of bacterial antibiotic resistance genes. Therefore, the key to ensuring that water quality is sufficient for human health is to develop an efficient, durable, low-cost and pollution-free water treatment material to address the aforementioned problems.

Here, we aimed to prepare a relatively safe and efficient treatment material that addresses some of the problems associated with the above treatment methods. In our previous study, the use of chitosan-modified biochar resulted in a superior treatment effect of drinking water^12^. Biochar is one of the most widely used materials in water treatment, as it is low-cost, renewable and highly porous and has high activity on its surface^13,14^. Biochar can be used to purify water by filtering impurities and adsorbing heavy metals and organic pollutants. For example, Trakal et al. used magnetically modified biochar to adsorb lead and cadmium in water^15^; Zazycki et al., extracted biochar from pecan nut shells and used it as a substitute sorbent to remove Active Red 141 from aqueous solutions at lower cost^16^. In addition, the combination of biomaterials is a promising area of research. For instance, Wang et al. prepared ZnO/biochar nanocomposites stabilized by carboxymethyl cellulose to enhance adsorption and photocatalytic degradation of methylene blue^17^. Yuanji Shi et al. used chitosan-modified biochar to remove dissolved organics matter from biotreated coking wastewater^18^. However, biochar itself does not kill microorganisms in large quantities and is not effective for long periods. Silver (Ag) is used as an antibacterial agent in water treatment, medicine, food packaging and other industries for its excellent antibacterial durability and broad-spectrum bacteriostasis. Diana Vilela et al. used Ag nanoparticles to create a self-propelled microrobot that could remove *Escherichia coli* from water^19^; Sayan Ganguly et al. used Ag nanoparticles to modify graphene oxide to generate bifunctional nanomaterials for catalysis and bacterial inhibition^20^. To maintain antibacterial efficacy, Ag requires a supporting matrix to sustain its morphological characteristics^21^, and biochar can play this role. Therefore, this complementary effect makes the highly porous silver biochar effective in killing microorganisms in water.

However, the shedding of silver leads to a reduction in its antibacterial ability and an excess of silver ions in water, which is a problem in the field of antibacterial water treatment. Moreover, the high silver loading leads to significant increases in the cost of antibacterial materials. Powdered biochar is difficult to apply directly in the water treatment process due to pressure drop. An effective way to solve the aforementioned problems is to first form a covalent bond between carbon and silver and then form a bead-shaped gel composite with C–Ag and polymer.

PVA is a type of polymer gel that is bio-friendly, non-toxic, water-soluble, biodegradable and biocompatible^22^. Its internal network structure and high mechanical strength and chemical stability following cross-linking provide a carrier that mediates the bacteriostatic effect of silver-bearing biochar in water^23^. However, PVA internal pores are relatively dense, which is not conducive to the loading and release of antimicrobial agents. To address this problem, calcium carbonate (CaCO_3_) can be added to the PVA during the material preparation process (as was done in this study), and the CaCO_3_ can be eluted during the subsequent processing so that the porosity of the PVA increases and the contact area with water increases, thereby enhancing the antibacterial effect. At the same time, calcium carbonate can bind metal ions with smaller radii, such as Cu^2+^ and Pb^2+^^24,25^. Therefore, the addition of calcium carbonate also enhances the heavy metal adsorption capacity to bacteriostatic material. Compared with our previously prepared water treatment membrane^26^, the material in this study has a particle morphology and additional heavy metal adsorption capacity, which provides a reasonable solution for the heavy metal pollution problems faced during water treatment and increases the applicability of this technique for water purification.

Here, we synthesized a porous polyvinyl alcohol/biochar polymer gel composite (p-PVA/C–Ag) using a simple and green method. Specifically, we used Fourier transform infrared spectroscopy (FT-IR) and X-ray diffraction (XRD) to determine the internal structure and elemental composition of the composite. We characterized the surface morphology of the material by scanning electron microscopy (SEM). We also tested the thermal stability of p-PVA/C–Ag with thermogravimetry–differential scanning calorimetry (TG-DSC). We used composite materials to conduct bacteriostatic experiments on *E. coli*. We studied application doses of composite materials, bacteriostatic persistence and reusability and discussed the antibacterial effects in simulated polluted water. Lastly, we analyzed the antibacterial mechanism of p-PVA/C–Ag particle antibacterial composites.

## Experimental

### Materials and reagents

All chemicals used in the study were of analytical grade. Polyvinyl alcohol was purchased from Guangzhou Jinhuada Chemical Reagent Co., Ltd. China. Boric acid, calcium carbonate (CaCO_3_) and ethanol absolute were purchased from Tianjin Tianli Chemical Reagent Co., Ltd. China. Sodium carbonate anhydrous (Na_2_CO_3_) was obtained from Tianjin Fuchen Chemical Reagent Factory in China. Silver nitrate (AgNO_3_) was purchased from Shanghai Shenbo Chemical Industry Co., Ltd. China. Copper(II) nitrate trihydrate (Cu(NO_3_)_2_·3H_2_O) was provided by Tianjin Bodi Chemical Co. Ltd. China. Lead nitrate (Pb(NO_3_)_2_) was supplied by Tianjin Dengfeng Chemical Reagent Factory in China. The corn straw was obtained from local farmers in Shaanxi Province in China. The model bacteria *E. coli* were purchased from the Microbiology Institute of Shaanxi in China.

### Preparation of C–Ag

The preparation process used the following methods described by one of our previous studies^12^. Briefly, corn straw (5 g) was dipped into 200 mL of 0.1 mol L^−1^ silver nitrate solution for 24 h and then dried completely. The silver nitrate-impregnated straw was then placed in a tube furnace for high-temperature carbonization. The tube furnace was heated to a temperature of 900 °C at a rate of 10 °C min^−1^ and under a nitrogen atmosphere for 1 h. Finally, the tube furnace was gradually cooled to room temperature, and the straw was ground (60-mesh sieve) and collected.

### Preparation of p-PVA/C–Ag

First, 1.5 g of PVA was added to 25 mL of deionized water with continuous stirring at 90 °C for 1 h. Next, 0.1 g of C–Ag was weighed and added into the PVA solution, and stirring was continued until the mixture was homogeneous. The calcium carbonate dissolved in absolute ethanol was poured into the mixed solution (1 g of calcium carbonate powder dissolved in 10 mL of absolute ethanol), and heating and stirring continued until the ethanol was completely evaporated. Next, 0.075 g of boric acid was added for crosslinking for 30 min. The aforementioned mixture was dropped into a 10% sodium carbonate solution and coagulated for 20 min. The excess sodium carbonate was washed off the obtained gel beads with deionized water and then freeze-dried. The lyophilized pellets were immersed in a 2 mol L^−1^ hydrochloric acid solution until no air bubbles were generated. The pellet was taken out and washed with deionized water to neutrality and then freeze-dried again to obtain the porous PVA/C–Ag composite material, which was abbreviated as p-PVA/C–Ag.

PVA/C–Ag without calcium carbonate and PVA (carbonate)/C–Ag without elution were prepared by a similar method, and they were noted as PVA/C–Ag and PVA(Ca)/C–Ag, respectively.

### Characterization of p-PVA/C–Ag

The chemical compositions of the materials were analyzed using a Nicolet iS 50ATR infrared spectrometer (Thermo Scientific Co., USA) in the range of 500–4000 cm^−1^. An X-ray diffractometer (XRD, Bruker D8, Germany) was used to analyze the crystal structure and bonding state of Ag in the composites. The angle range of scanning was from 10° to 80°. The surface morphologies of composites were observed by scanning electron microscopy (SEM, SU8010, Hitachi, Japan). The relevant characteristics of the pores in the composite were tested by an Automatic Mercury Porosimeter (Autopore IV 9500, Micromeritics, USA). p-PVA/C-Ag was estimated by thermogravimetric and differential scanning calorimetry (TG-DSC, Labsys Evo, Setaram, France). The concentrations of Ag^+^, Cu^2+^ and Pb^2+^ in water were detected by a graphite furnace atomic absorption spectrophotometer (GFAAS, Thermo co., USA).

### Antibacterial testing and silver loss of p-PVA/C–Ag

Bacteria in the third generation of the *E. coli* cultivation were used for the bacteriostatic experiment. All glassware used in the experiment was sterilized in an autoclave at 121 °C for 20 min in advance. The bacteriostatic tests were conducted using the plate counting method and the inhibition zone method.

For more details on the antibacterial experiments, see our previous study^12^. The antibacterial ratio formula (1) was calculated as follows:

1 $$antibacterial ratio=\frac{{A}_{0}-A}{{A}_{0}}\times 100\%$$

where *A*_*0*_ is the initial number of colonies and *A* is the number of colonies after bacteriostasis.

To verify the antibacterial effect of the composite material at different doses and times, optimization experiments were conducted with *E. coli*. The original concentration of *E. coli* suspension in the plate counting method was 2.1 × 10^8^ CFU mL^−1^. The dosage of p-PVA/C–Ag composite was 0.2, 0.3, 0.4, 0.5, 0.6, 0.8 and 1.0 g L^−1^. The bacterial suspension was cultured in a constant temperature shaking incubator for 1, 3, 5, 7 and 9 h at 37°. Next, 0.1 mL of the suspension was measured and diluted with sterile water to an appropriate concentration to facilitate counts. All experiments were run in parallel in triplicate, and the average values were used in subsequent analyses.

Bacteriostasis of p-PVA/C–Ag composite was tested in a laboratory-simulated polluted water environment (2.1 × 10^5^ CFU mL^−1^). Simulated antibacterial tests were conducted in an adsorption column with a bed height of 18 cm and an inner diameter of 1.5 cm. Separate comparative tests with p-PVA/C–Ag and activated charcoal in the bed (4 mL) were conducted at a water flow rate of 2 mL s^−1^. The concentration of bacteria (*E. coli*) in the contaminated water was measured by the plate counting method.

The reusability of p-PVA/C–Ag particle composite is an important criterion for measuring the cost performance of materials. The p-PVA/C–Ag was rinsed with deionized water after one use and freeze-dried again (noted as p-PVA/C–Ag, U & F), and the inhibition test was performed again; the test was repeated several times according to this method.

Silver ions are one of the most important heavy metal ions threatening human health and the natural environment. National drinking water standards (GB5749-2006, China) place strict regulations on them, and the maximum Ag content in drinking water must be lower than 0.05 mg L^−1^. Therefore, the loss of silver ions from p-PVA/C–Ag composites in drinking water provides an important means for detecting safety hazards. The bacteriostatic material (0.06 g) were placed in 100 mL of drinking water to test silver losses in 30 consecutive days, and the concentration of Ag^+^ in solution was gauged using a graphite furnace atomic absorption spectrophotometer (GFAAS, Thermo Co., USA) every five days starting on the fifth day.

### Swelling ratio of p-PVA/C–Ag

The swelling ratio of water treatment materials is one of the most important indicators of their performance. Twenty dried p-PVA/C–Ag pellets prepared previously were weighed. They were placed in deionized water and removed from the water every 0.5 h. The surface water was wiped with filter paper, the mass was measured and the data were recorded. The formula (2) for the swelling ratio was as follows.

2 $$Swelling ratio=\frac{M-{M}_{0}}{{M}_{0}}\times 100\%$$

where *M* is the mass of the pellet at some point, and *M*_*0*_ is the mass of the initial dry pellet.

### Adsorption test (Cu^2+^, Pb^2+^) of p-PVA/C–Ag

Half a gram of p-PVA/C–Ag composite was placed in a 100-mL solution of Cu(NO_3_)_2_·3H_2_O and Pb(NO_3_)_2_ at a concentration of 100 mg L^−1^. The adsorption times were 0, 1, 3, 6, 8, 12, 23 and 24 h. The adsorption ratio was then calculated using the following formula (3):

3 $$adsorption ratio=\frac{{C}_{0}-C}{{C}_{0}}\times 100\%$$

where *C* is the concentration after adsorption for a certain period, and *C*_*0*_ is the initial concentration.

## Results and discussion

### Characterization of p-PVA/C–Ag composite

#### FTIR analysis

**Figure 1:**
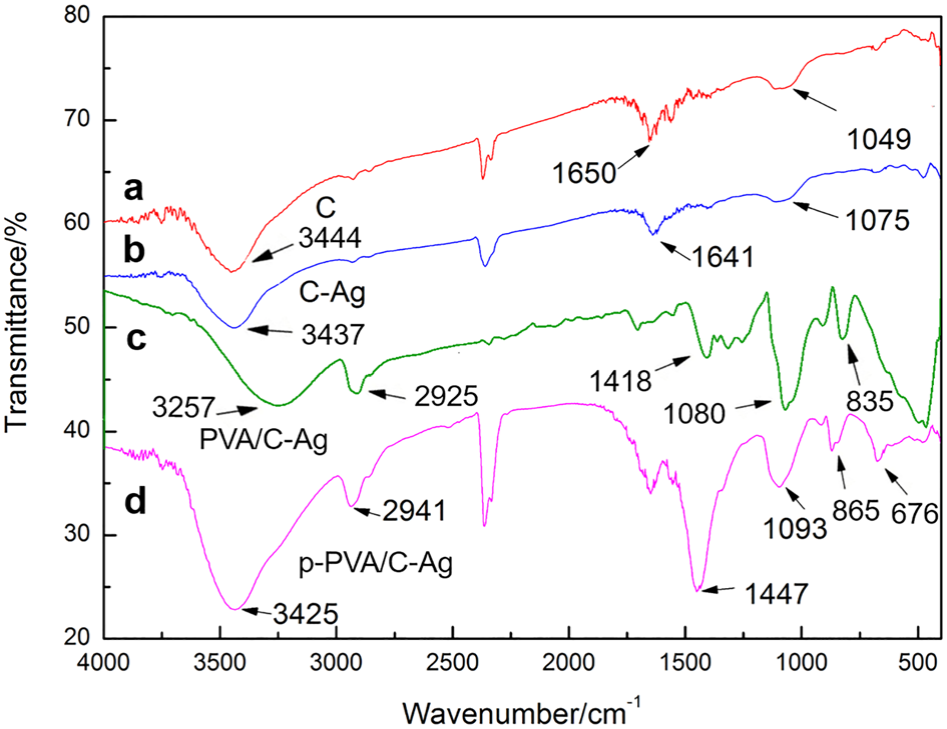
FT-IR spectrogram of (**a**) C900, (**b**) C–Ag, (**c**) PVA/C–Ag, (**d**) p-PVA/C–Ag.

Some organic functional groups were present in the corn stover biochar after carbonization: 3444 cm^−1^ corresponded to the O–H stretching vibration, and 1650 cm^−1^ corresponded to the C=C vibration (Fig. 1a). The intensity of peaks after carbonization was significantly weaker at the aforementioned two positions (Fig. 1b). In curve c, the stretching vibration peak of hydroxyl was shifted to 3437 cm^−1^, which might be attributed to the coordination of hydroxyl groups and silver. During carbonization, there was a large number of reducing organic functional groups in corn stalks, which reduced Ag^+^ to Ag particles. In curve d, 3425 cm^−1^ corresponded to the stretching vibration peak of O–H in p-PVA/C–Ag; 2941 cm^−1^ and 1447 cm^−1^ corresponded to the C-H stretching and bending vibration, respectively; and 1093 cm^−1^ corresponded to the bending vibration of C–O–C in PVA^27,28^.

Compared with PVA/C–Ag (Fig. 1c), the p-PVA/C–Ag (Fig. 1d) showed a much larger peak at 1447 cm^−1^, which indicates that a small amount of calcium carbonate remained in the material after elution with hydrochloric acid^29^. This type of calcium carbonate residue also provided certain benefits; for example, it will endow a certain adsorption capacity to the bacteriostatic material for Cu^2+^and Pb^2+^, which was also confirmed by subsequent experiments.

#### X-ray diffraction analysis

**Figure 2:**
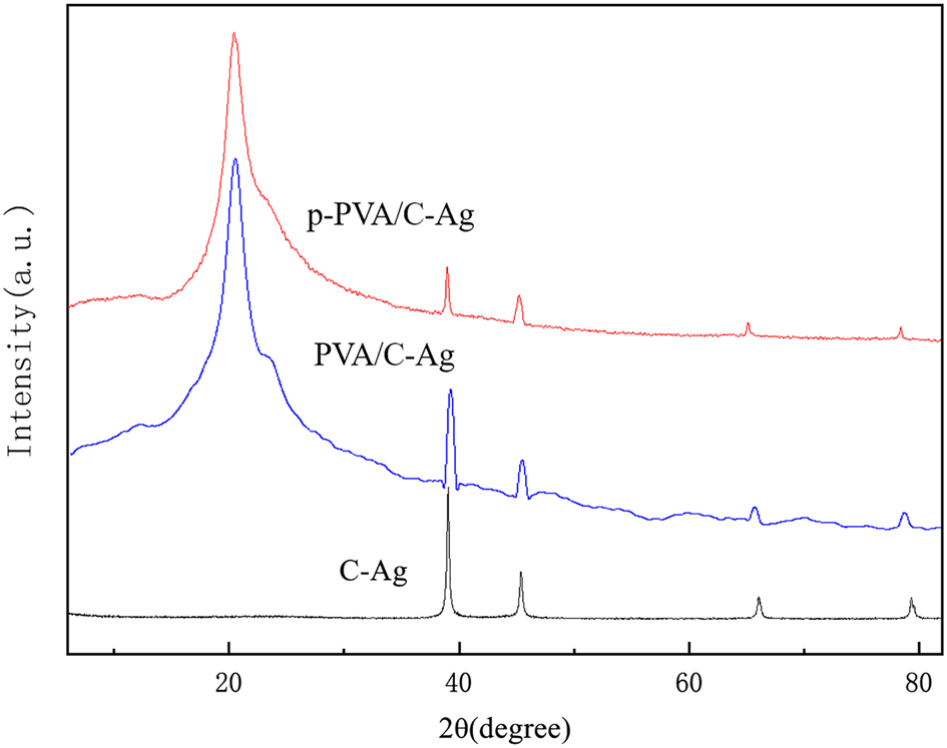
XRD pattern of C–Ag, PVA/C–Ag and p-PVA/C–Ag composite.

The diffraction peaks of silver in all materials were located at the same position (Fig. 2). The positions of the diffraction peak at 2θ = 38.18°, 44.34°, 64.46° and 77.40° were assigned to the (111), (200), (220) and (311) planes, respectively, which is consistent with the standard card of Ag (JCPDS No. 04-0783)^30,31^. The p-PVA/C–Ag was less intense than the other peaks, likely because silver was coated inside the PVA particles. At 2θ = 20°, the composite had a distinct peak, primarily because of the diffraction peak of the PVA crystal on the (101) crystal plane^32^, which also indicated that PVA was present in the p-PVA/C–Ag composite. The size of the silver particles in the composite material was calculated to be approximately 50 nm by the Scherrer formula.

#### SEM analysis

**Figure 3:**
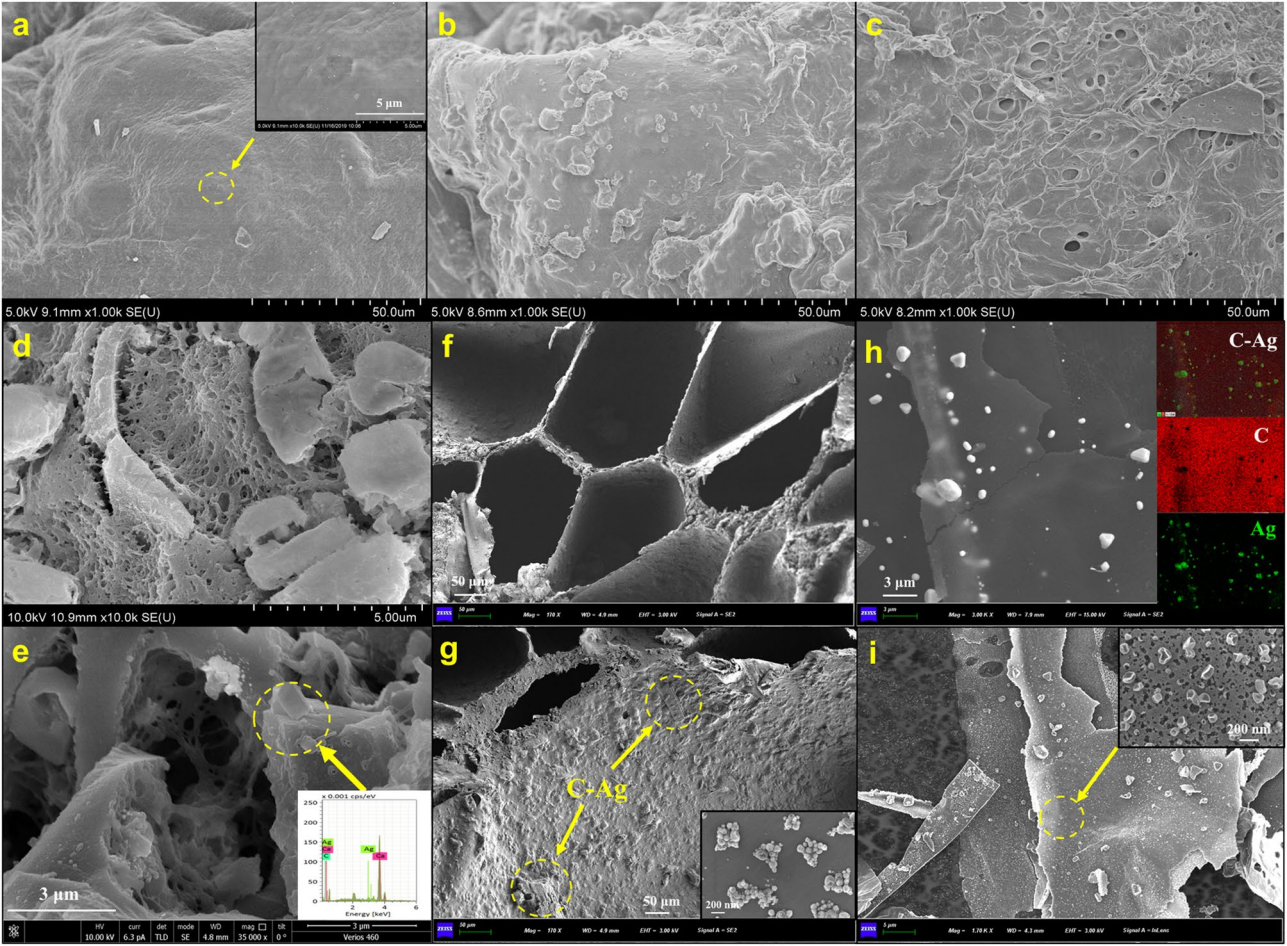
Surface morphology of (**a**) PVA/C–Ag, (**b**) PVA(Ca)/C–Ag, (**c**) p-PVA/C–Ag; (**d**) fracture surface of PVA/C–Ag, (**e**) p-PVA/C–Ag; (**f**,**g**) fracture surface of the original p-PVA/C–Ag and the material after use and freeze-drying; (**h**) SEM and mapping images of C–Ag; (**i**) SEM images of C–Ag (inset shows the Ag nanoparticles on the surface of C–Ag).

To demonstrate the structural superiority of p-PVA/C–Ag, three different materials were compared by SEM. Figure 3a–c show SEM images of the surface morphology of PVA/C–Ag, PVA (Ca)/C–Ag and p-PVA/C–Ag, respectively. With the elution of calcium carbonate, many holes appeared on the exterior surface of p-PVA/C–Ag (Fig. 3c); by comparison, holes on the surfaces of the other two materials were dense. The inset of Fig. 3a shows that the flake biochar was wrapped in PVA. There were high numbers of micropores on PVA/C–Ag, but p-PVA/C–Ag had many macropores aside from micropores, as some fragmented particles and some crystalline substances can also be seen (Fig. 3d,e). These particles were caused by fragmented silver-loaded biochar coated in PVA gel, and the crystals may stem from small amounts of residual calcium carbonate. The EDS (the inset of Fig. 3e) of p-PVA/C–Ag also confirmed the existence of the above materials. In the process of high-temperature carbonization, silver ions were reduced to Ag nanoparticles by the reducing functional groups in corn straws. SEM of C–Ag as well as mapping results (inset of Fig. 3g,h) demonstrate the presence of silver nanoparticles. The porous structure of the polymer material facilitates the absorption of bacteria and the release of the bacteriostatic agent, thereby enhancing the bacteriostatic effect. The calcium carbonate residue thus permits the material to adsorb heavy metals. The shape of the pores of p-PVA/C–Ag did not change significantly before and after application (Fig. 3f,g). Figure 3i shows the sheet structure of C–Ag with an inset that clearly demonstrates the formation of silver nanoparticles on the carbon sheet.

**Figure 4:**
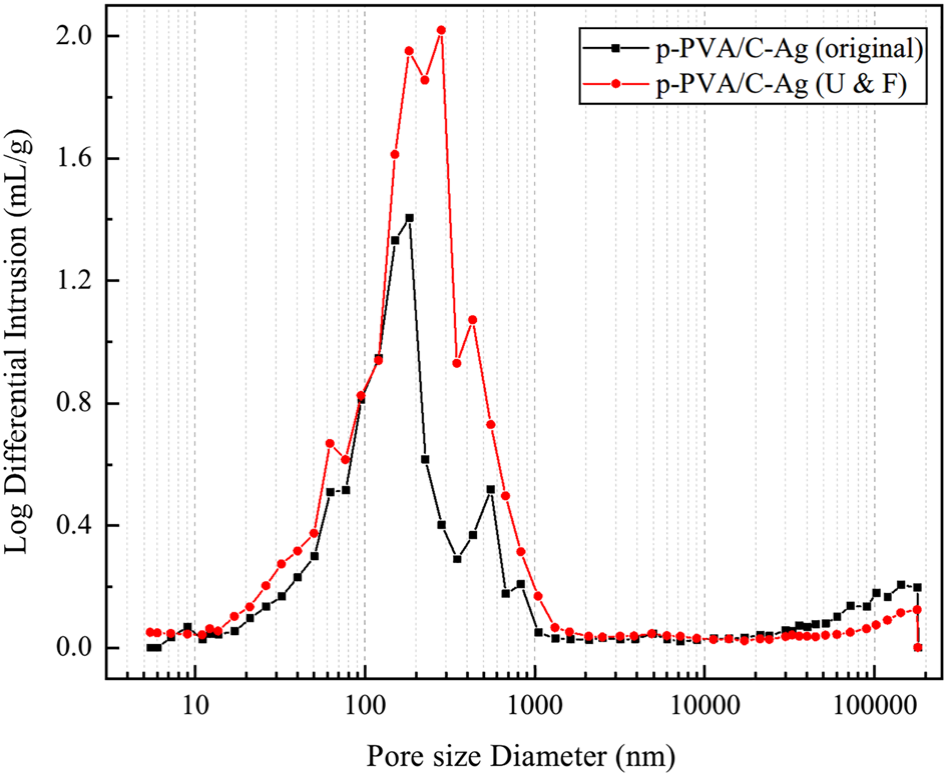
Pore size distribution of the original p-PVA/C–Ag and the material after use and freeze-drying.

With the aid of an Automatic Mercury Porosimeter, the relevant characteristics of the pores in the composite were also tested. The pore sizes in the original p-PVA/C–Ag were mainly concentrated between 80 and 1000 nm (Fig. 4), with an average pore diameter (4 V/A) of 116.5 nm. The pore size distribution remained approximately the same after the material was used and freeze-dried again, and an increase in the amount of incoming mercury in the graph indicated that the total volume of the pores increased. The test results showed that the average pore size of p-PVA/C–Ag (U & F) increased to 123.2 nm, and the porosity increased from 59.74 to 69.99%. During the use of the material, the residual calcium carbonate was further removed by prolonged soaking and elution as well as the continuously released of silver particles, which resulted in a slight increase in porosity. However, the main reason for the change in pore size is that several hours of immersion and washing filled the pores of p-PVA/C–Ag with water, and the volume expansion of water after freezing and icing caused the internal pores to be propped up; freeze-drying allowed this structure to be retained, eventually leading to larger porosity.

#### TG and DSC analysis

**Figure 5:**
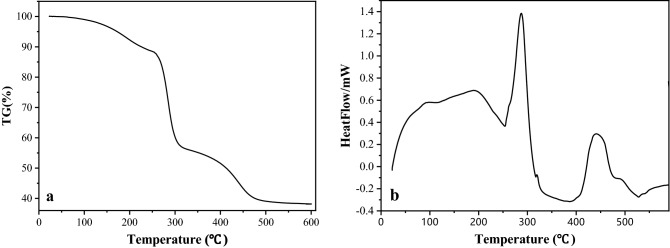
(**a**) TG curve of p-PVA/C–Ag composite; (**b**) DSC curve of p-PVA/C–Ag composite.

The TG and DSG curves of p-PVA/C–Ag composites from 25° to 600° are shown in Fig. 5. The process of weight loss can be classified into three main stages. Stage 1 was from 100 to 250 °C, and the weight loss was approximately 12%; weight loss primarily stemmed from the evaporation of the combined water in the composite, which was reflected by an endothermic peak^33^. The second stage was from 250 to 310 °C, and the weight loss was approximately 30%; weight loss primarily stemmed from the decomposition of side chains in the PVA polymer and was reflected by an endothermic peak^34^. The last stage was from 310 to 530 °C, where the breakage of the C–C bonds in PVA polymers resulted in 20% weight loss^35^. Finally, the TG curve remained stable, with a weight of approximately 38%, and the main components were C–Ag, residual CaCO_3_ and carbonized PVA. Therefore, p-PVA/C–Ag particle composite possessed high stability during the drinking water treatment process.

### Performance of p-PVA/C–Ag composite

#### Antibacterial activity of p-PVA/C–Ag composite

**Figure 6:**
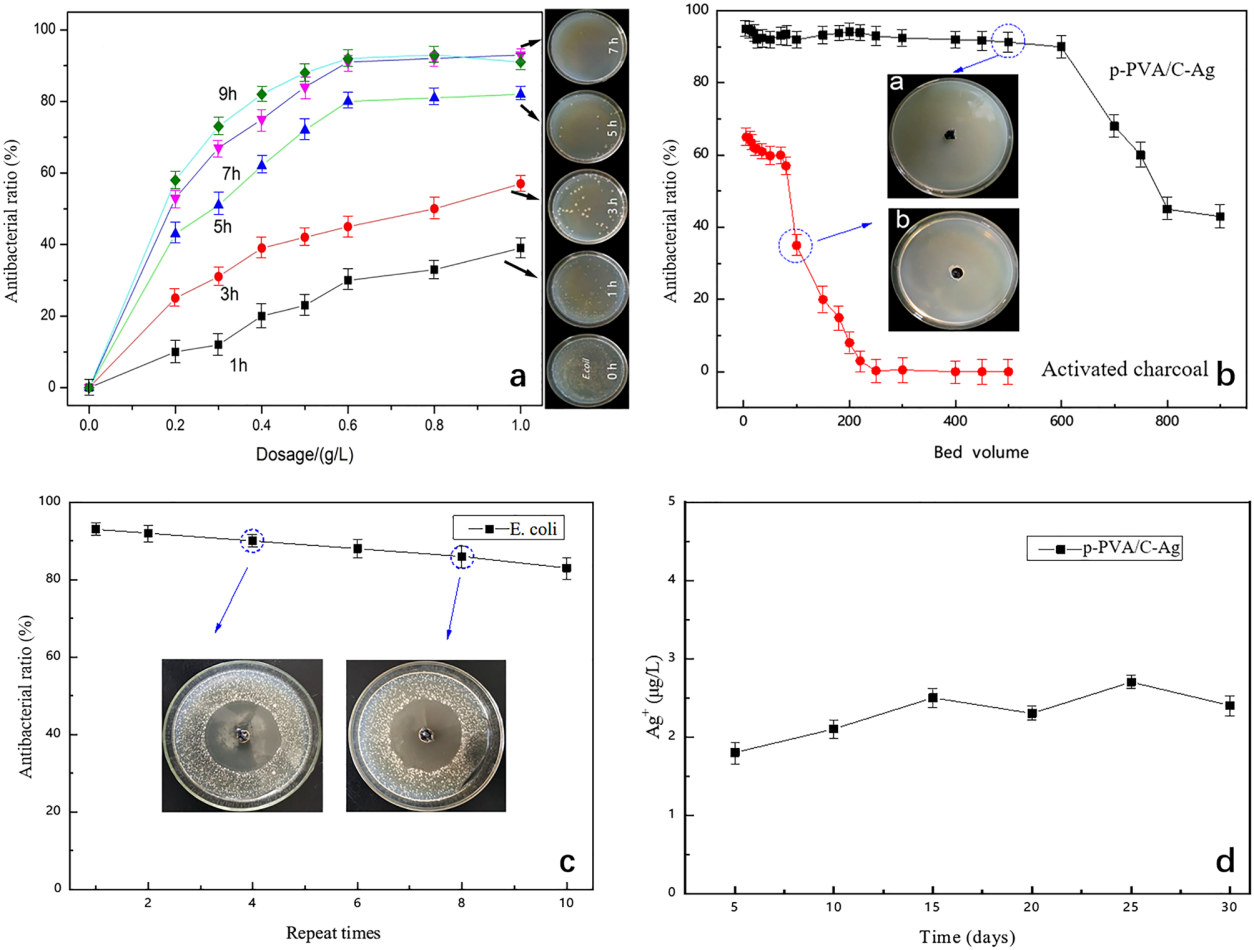
(**a**) Dose and time optimization of p-PVA/C–Ag composite; (**b**) bacterial inhibition effect of p-PVA/C–Ag composite on simulated actual polluted water; (**c**) reusability of p-PVA/C–Ag composite; (**d**) silver release of p-PVA/C–Ag composite.

The bacteriostatic effect of p-PVA/C–Ag composites changed as the dose and bacteriostatic time increased (Fig. 6a). The figure shows the inhibition of *E. coli* (the initial concentration of the *E. coli* suspension was 2.3 × 10^8^ CFU mL^−1^) at different times (0, 1, 3, 5, 7 and 9 h) when the compound was added at a dosage of 0.6 g L^−1^. When the amount added was 1 g L^−1^, the bacteriostatic effect was not greater than 0.6 g L^−1^, and the number of bacteria after 7 h of bacteriostasis hardly changed. The p-PVA/C–Ag composite had the strongest bacteriostatic effect when the dosage was 0.6 g L^−1^ and the inhibition time was 7 h, and the inhibition ratio was greater than 90%. In addition, we tested p-PVA/C–Ag against *Staphylococcus aureus* as well as *Pseudomonas aeruginosa* (see Supplementary Fig. S1 online); the results showed that the composite had a significant inhibitory effect on both Gram-positive and Gram-negative bacteria.

The p-PVA/C–Ag composites maintained over 90% inhibition until 600 column volumes were filtered in simulated actual polluted water (Fig. 6b). When the column volume was greater than 600, the bacteriostatic effect decreased, indicating that the composite had lost some antibacterial ability. The bacteriostatic ratio of activated carbon decreased significantly after filtering less than 100 column volumes, and it soon lost its bacteriostatic ability, which was primarily related to its adsorption effect. The inset of Fig. 6b shows the bacterial inhibition capacity of p-PVA/C–Ag composites after filtration of the 500th column volume (up) and activated carbon after filtration of the 100th column volume (bottom), respectively. The bacteria grew around the antibacterial active carbon, indicating that the active carbon itself had no actual bactericidal effect. However, the p-PVA/C–Ag composite material still had a high sterilization effect after filtering 500 column volumes, and its surroundings appeared aseptic, which further suggested that the composite material had a better sterilization ability.

The bacteriostatic tests showed that p-PVA/C–Ag had high reusability. Figure 6c clearly shows that after the composite material was reused 10 times, the bacteriostatic ratio was still 80%. The inset shows that after 4 and 8 times of reuse, there was an obvious inhibition zone. After each reuse, the bacteriostatic performance of the composite material decreased, primarily because further inhibition was hindered by some of the killed bacteria that remained inside and on the surface. After multiple releases, the Ag content in the material gradually decreased, resulting in a small decrease in bacteriostatic properties. Figure 6d shows the silver release of the p-PVA/C–Ag composite after repeated use. The silver ions in the water were less than 5 μg L^−1^ after 30 days of continuous monitoring, which is far below the national standard for drinking water (GB5749-2006, China), indicating that p-PVA/C–Ag is safe and reliable to use.

#### Physical properties of p-PVA/C–Ag composite

**Figure 7:**
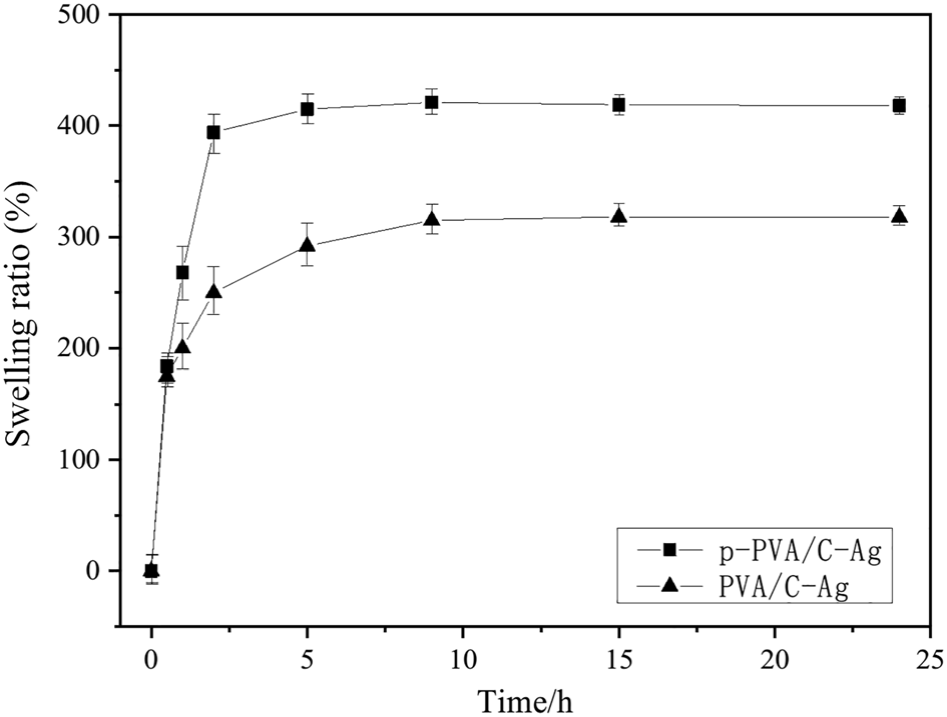
Swelling ratio of p-PVA/C–Ag and PVA/C–Ag composite.

In practice, the swelling of the material provides an important reference point. The swelling ratio of the material increased as the soaking time increased over a certain time frame (Fig. 7). When the immersion time reached approximately 7 h, the swelling equilibrium was reached. The swelling ratio of the p-PVA/C–Ag particles was approximately 400%, and the diameter of the beads was approximately 2 mm. By comparison, the swelling ratio of PVA/C–Ag at equilibrium was 20.5% lower (only ca. 318%). The reduced swelling ratio can be explained by the higher abundance of water in the macropores in p-PVA/C–Ag. In the water environment, the compressive capacity of the particles after full swelling provided another important indicator that can be used to assess the mechanical properties of the particles. The fully swelled particles were subjected to compression testing using an electronic universal testing machine. The results indicated that the maximum load of the particles was 25.3 ± 0.2 N, further illustrating that the p-PVA/C–Ag particles show high potential for application as a packing agent for the adsorption column in water treatment.

#### Adsorption capacity of PVA/C–Ag composite (Cu^2+^, Pb^2+^)

**Figure 8:**
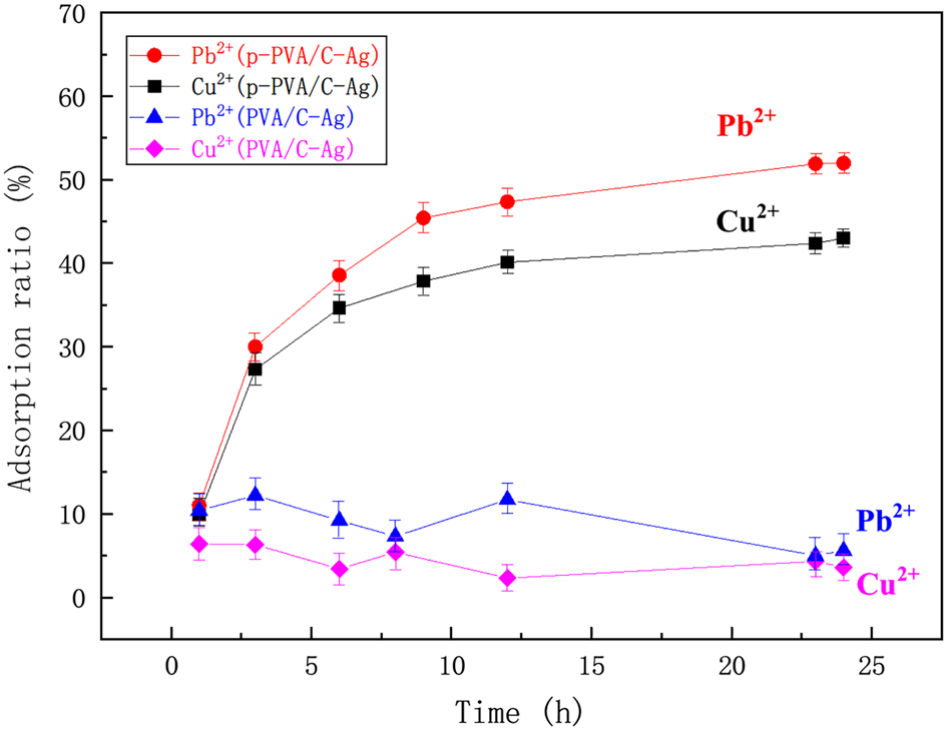
Adsorption effect of PVA/C–Ag composite on Cu^2+^ and Pb^2+^.

The experimental results of the adsorption of metal ions (Cu^2+^ and Pb^2+^) in polluted water are shown in Fig. 8. The adsorption ratio of p-PVA/C–Ag composite for Cu^2+^ and Pb^2+^ were 43% and 51%, respectively. In contrast, PVA/C–Ag absorbed little Cu^2+^ and Pb^2+^. Therefore, the composite could adsorb both metal ions. This adsorption capacity stemmed from the small amount of calcium carbonate residue in the material and also confirmed that the previous FTIR and SEM analyses were robust. Therefore, the p-PVA/C–Ag composite material had possessed strong antibacterial properties and a high adsorption effect.

#### Synergistic mechanism of bacterial inhibition by p-PVA/C–Ag

**Figure 9:**
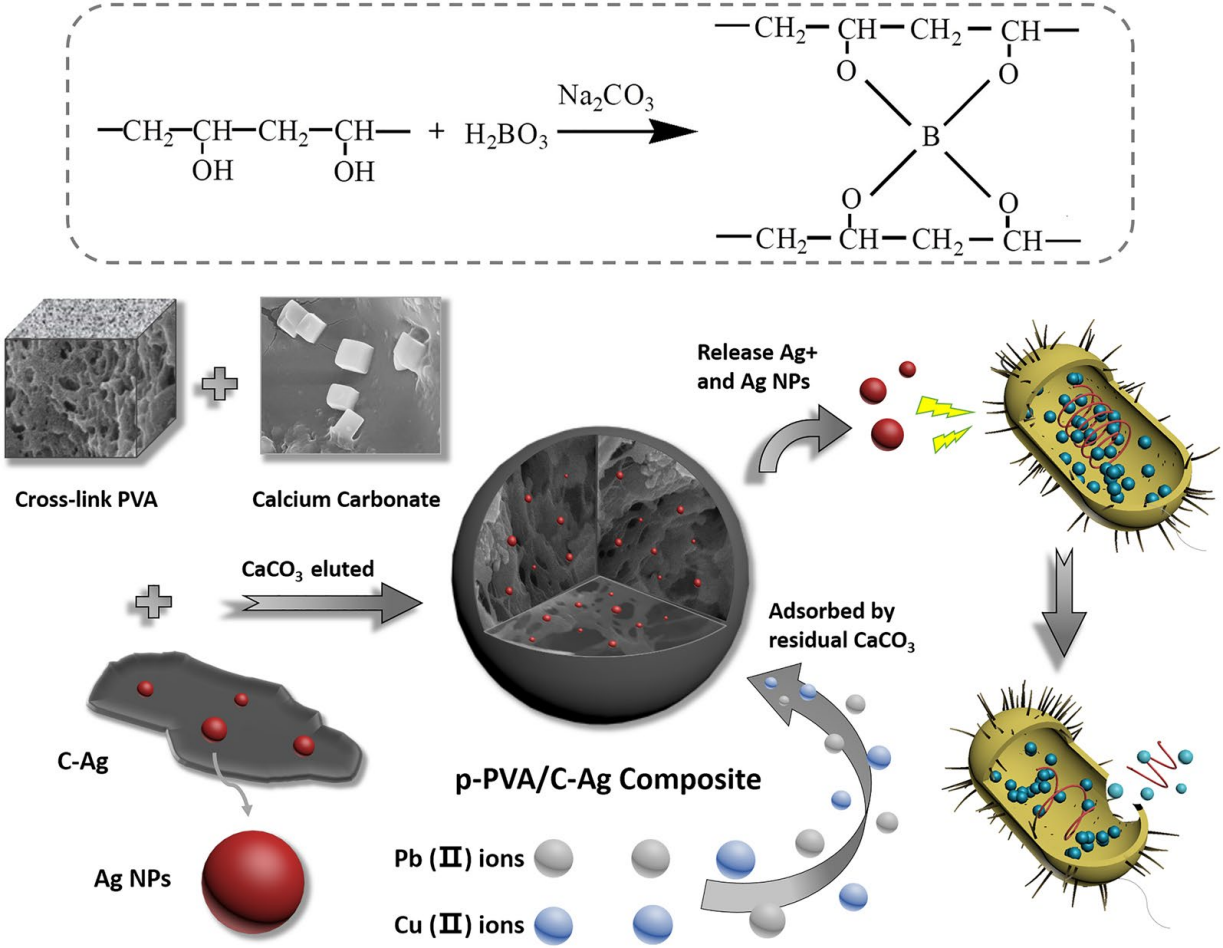
Schematic diagram of the antibacterial mechanism of p-PVA/C–Ag composite.

PVA is a reticulated polymer gel with a cross-network structure that provides a carrier for dispersing silver-loaded biochar (C–Ag) during the bacteriostatic action of p-PVA/C–Ag composite. At the same time, it can swell after being immersed in water because of its gel network structure, permitting the bacteriostatic material to fully contact with the water environment and have a continuous sterilizing effect. The biochar in the C–Ag is derived from the carbonization of corn straw at high temperature, and the carbonized biochar has a specific surface area of 700 m^2^ g^−1^^12^. The porous structure and large specific surface area of biochar also provide suitable conditions for loading silver. Biochar after silver loading has a strong bactericidal effect because of the wide and persistent bactericidal ability of silver. The addition and elution of CaCO_3_ make the macropore structure appear in PVA. The large surface area and pores provide high adsorption performance, which can adsorb bacteria and other microorganisms on the surface and inside of the gel material. Silver-loaded biochar can exert a specific antibacterial effect via its dispersal in the reticular structure of the PVA gel (Fig. 9). Ag^+^ and nano-silver are the major inhibitors of bacteria in the composite material. Ag^+^ cross the cell membrane and enter the cytoplasm, thus destroying the internal structure of the bacteria^19^. Ag NPs can also pass through bacterial cell walls and inactivate bacteria^20^. Consequently, most of the *E. coli* is adsorbed and retained in the pores of the PVA. The large contact area provides a platform for the interaction of bacteria and bacteriostatic agents and further promotes the destruction of microorganisms. In addition, the residual CaCO_3_ in p-PVA/C–Ag gives the composite the ability to adsorb copper and lead.

## Conclusion

Ag was loaded on biochar using high-temperature carbonization reduction. High-porosity p-PVA/C–Ag composite was synthesized and combined with polymer PVA gel. FTIR and XRD characterization confirmed the successful preparation of the material. The SEM images clearly demonstrated the existence of micropores and macropores in the material, which explain the high porosity of the composite, and the nano-silver particles were evenly distributed on the biochar and PVA. TG-DSC showed that the polymer material had high thermal stability. The results of the bacteriostatic tests showed that the composites greatly inhibited *E. coli*. p-PVA/C–Ag gel with high swelling performance also showed higher performance in tests of reusability, bacteriostatic persistence and the simulated treatment of polluted water. The amount of silver lost from p-PVA/C–Ag in water was far below the standards for drinking water quality (GB5749-2006, China). In addition, the residual CaCO_3_ in PVA could adsorb heavy metal ions, such as Cu^2+^ and Pb^2+^. In sum, this cost-effective, safe and reliable material shows much potential to be used in the purification of drinking water and related applications.

## Supplementary Information

Supplementary Information 1.
