# Supplementary material for: Preparation and bacteriostatic research of porous polyvinyl alcohol / biochar / nanosilver polymer gel for drinking water treatment
Source: Sci Rep. 2021 Jun 9;11:12205. doi: 10.1038/s41598-021-91833-9 (PMC8190314; doi:10.1038/s41598-021-91833-9)
Supplement: Supplementary file 1 — Supplementary Information 1. [file 41598_2021_91833_MOESM1_ESM.pdf]

Supplementary Information

Preparation and Bacteriostatic Research of Porous Polyvinyl Alcohol / Biochar / Nanosilver Polymer Gel for Drinking Water Treatment

Liang Zhang[a,b*](zl98zl@hotmail.com), Hang Zhao[a], Zhihui Hu[a], Lvling Zhong[a], Juanqin Xue[a]

[a] School of Chemistry and Chemical Engineering, Xi’an University of Architecture and Technology, Xi’an, Shaanxi, 710055, China

[b] Shannxi Provincial Key Laboratory of Gold and Resource, Xi'an University of Architecture and Technology, Xi'an 710055, Shaanxi, China

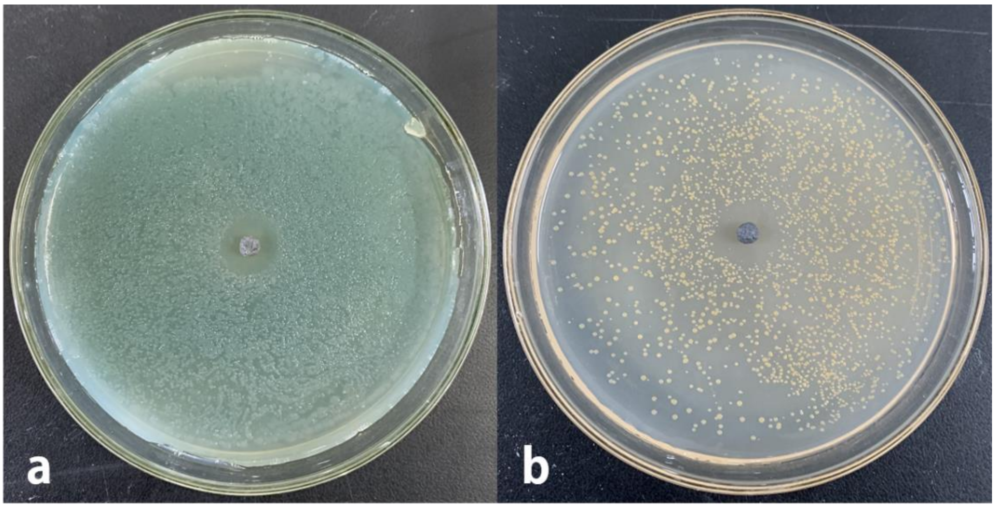

Figure S1. Bacterial inhibition test results of p-PVA/C-Ag against *P. aeruginosa* (a) and *S. aureus* (b)
